# Partnered sexual activity and satisfaction in women with spinal cord disease: influence of urinary incontinence and other predictors

**DOI:** 10.1038/s41393-026-01214-0

**Published:** 2026-05-13

**Authors:** Rachel Mazoni Costa, Manuela Horta, André Tunes de Paula, Taynna Ferreira Arantes da Costa, José de Bessa, William Carlos Nahas, Cristiano Mendes Gomes

**Affiliations:** 1 Division of Urology, Universidade de São Paulo, São Paulo, Brazil; 2 Department of Urology, Hospital Evangélico de Belo Horizonte, Belo Horizonte, Minas Gerais Brazil; 3 Division of Urology, Universidade Estadual de Feira de Santana, Feira de Santana, Bahia Brazil

**Keywords:** Urological manifestations, Quality of life

## Abstract

**Study design:**

Cross-sectional study.

**Objectives:**

Evaluate sexual activity and satisfaction in women with spinal cord disease (SCD) and their associations with urinary incontinence.

**Setting:**

Outpatient neuro-urology clinics at a tertiary rehabilitation center in Brazil.

**Methods:**

Ninety-eight women aged ≥18 years with traumatic or non-traumatic SCD were included. Clinical and demographic data were collected through structured interviews. Bladder symptoms were assessed with the Neurogenic Bladder Symptom Score–Short Form (NBSS-SF). Sexual function was evaluated with the Female Sexual Function Index (FSFI), and sexual satisfaction with the WHOQOL-BREF. Sexual activity was defined as partnered sexual activity, with or without intercourse, within the prior six months. Logistic regression identified predictors of sexual activity and satisfaction.

**Results:**

Mean age was 42.9 (±12.1) years; most cases were non-traumatic (85.7%), mainly multiple sclerosis (55.9%). Urinary incontinence was reported by 52%, severe in 28.6%. Sexual activity was reported by 48 women (49.0%), of whom 58.3% had FSFI-defined dysfunction and 60.4% reported satisfaction. Incontinence was strongly associated with inactivity (72.5% vs. 27.5%, p < 0.001) and lower FSFI scores (16.2 (10.3) vs. 24.4 (9.1), p < 0.001). Urinary continence (OR 5.0, 95% CI, 1.7 to 14.4), younger age (OR 0.93/year, 95% CI, 0.89 to 0.98), and being married (OR 10.9, 95% CI, 3.6 to 33.3) predicted sexual activity and satisfaction.

**Conclusions:**

Sexual dysfunction and UI were prevalent and interrelated in women with SCD. UI independently predicted inactivity and dissatisfaction.

## Introduction

Spinal cord disorders (SCD), including both traumatic spinal cord injury (SCI) and non-traumatic etiologies such as multiple sclerosis, transverse myelitis, and myelomeningocele, affect multiple domains of quality of life. In women, these conditions often disrupt sexual health through a combination of neurophysiological, psychological, and relational mechanisms [1–3]. Sexual dysfunction in women with SCD may present as reduced desire, impaired arousal, diminished vaginal lubrication, difficulty achieving orgasm, and overall dissatisfaction with sexual life [4, 5], often coexisting with impaired mobility, sensory deficits, fatigue, altered body image, and mood disorders [6, 7].

Lower urinary tract dysfunction (LUTD), particularly urinary incontinence (UI), is one of the most distressing comorbidities in this context. UI may lead to embarrassment, avoidance of intimacy, and diminished self-esteem [8–10], while bladder management strategies such as intermittent catheterization or use of absorbent products can further affect confidence and compromise sexual well-being [11, 12].

Despite the high prevalence of both neurogenic LUTD and sexual dysfunction in women with SCD [8, 13], their shared neuroanatomical pathways, and the international guidelines and consensus documents recommending routine assessment and counselling regarding lower urinary tract symptoms and sexual function in individuals with spinal cord injury or spinal cord disease, data on sexual activity and its clinical predictors in women remain limited [13, 14].

A clearer understanding of how LUTD intersects with sexual activity and satisfaction in women with SCD is necessary to guide targeted rehabilitation strategies. Identification of potentially modifiable contributors, including urinary incontinence, may yield actionable opportunities to improve quality of life in this vulnerable population [9, 14–16].

The aim of this study was to evaluate sexual activity and satisfaction in women with SCD and to investigate their associations with urinary incontinence and other clinical and demographic predictors of sexual function.

## Methods

This cross-sectional study evaluated the sexual function and related factors in women with spinal cord diseases (SCD) of both traumatic and non-traumatic etiologies. Participants were recruited during routine medical visits to the outpatient neurology, urology and spinal cord disease clinics of a tertiary rehabilitation centre. This interdisciplinary program includes neurology, physiatry, urology, nursing, psychology, and pelvic floor physiotherapy. Routine care involves structured management of LUTD (including urological assessment and treatment), education on bladder and bowel routines, and, when appropriate, counselling on sexual function and reproductive health. The primary objective was to assess how urinary incontinence and bladder management impact sexual life in women living with SCD. The study was approved by the Institutional Ethics Board (IRB Approval N° 86965218.3.0000.0068), and informed consent was obtained from all participants.

The study enrolled women aged 18 years or older with SCD for at least one year and sufficient cognitive ability to understand and consent were eligible for the study. Patients with language barriers or cognitive impairment that prevented adequate comprehension of the study materials were excluded. We investigated factors associated with sexual activity, satisfaction with sexual life, and the interplay between sexual outcomes, bladder management, and incontinence severity.

Clinical and sociodemographic data (age, marital status, education, and occupation), SCD-related characteristics (etiology, disease duration, and severity of neurological impairment), and general aspects of sexual life (sexual orientation and reasons for sexual inactivity) were obtained through in-person interviews conducted by trained investigators using a structured evaluation designed specifically for this study. Due to the heterogeneous etiologies, we did not apply a single standardized neurological scale. Instead, we used ambulatory status (walking independently, walking with assisted device/orthosis or wheelchair user) as a pragmatic and clinically relevant proxy for neurological impairment.

The Neurogenic Bladder Symptom Score – Short Form (NBSS-SF), a validated tool for neurological lower urinary tract dysfunction, was employed to assess bladder management and incontinence. The NBSS-SF scores range from 0 (best) to 28 (worst) and include subdomains such as incontinence, storage, voiding, and consequences. Satisfaction with bladder condition was defined as a response of “pleased” or “mostly satisfied” to the overall bladder-related quality of life question. Women reporting urine leakage a few times per week, or more were classified as incontinent, while severe incontinence was defined as daily leakage requiring ≥3 pads/day [17].

Sexual function was assessed using the Female Sexual Function Index (FSFI), a validated questionnaire that evaluates six domains: desire, arousal, lubrication, orgasm, satisfaction, and pain/discomfort. For the purposes of this study, sexual activity (with or without intercourse) was defined as any partnered sexual activity within the previous 6 months and was used for group classification. A total FSFI score of ≤26.55 was used as the validated screening cut-off to indicate that a woman’s sexual function fell within the dysfunctional range [18]. Women who reported no partnered sexual activity in the past six months were classified as sexually inactive; following common practice in FSFI studies, only their Desire domain scores were analysed, and total scores and other domain scores were not interpreted [19–21].

Satisfaction with sexual life was assessed using Question 21 of the WHOQOL-BREF (*“How satisfied are you with your sexual life?”)*, which provides a more direct, global assessment of sexual satisfaction that does not depend on recent sexual activity or specific sexual behaviour and minimizes the potential contextual bias of q16 of FSFI being embedded within a sexuality-focused questionnaire. For the purposes of this study, responses of “satisfied” or “very satisfied” were considered indicative of sexual satisfaction. Although the full WHOQOL-BREF was administered, only data from Question 21 were analyzed in this manuscript [22].

Univariate analyses were conducted to identify predictors of sexual activity and satisfaction. Variables with p < 0.10 were entered into multivariable logistic regression models to determine independent predictors. Associations between categorical variables were expressed as odds ratios (ORs) with 95% confidence intervals (CIs). Continuous variables were summarized as mean ± standard deviation or median (interquartile range), and compared using Student’s t-test or Mann–Whitney test, as appropriate. Categorical variables were analyzed with the chi-square test, and ANOVA with Bonferroni correction was applied for comparisons across more than two groups. Correlations were evaluated using Spearman’s coefficient. Statistical significance was set at p < 0.05. Analyses were performed using GraphPad Prism, version 5.0.3 (GraphPad Software, San Diego, CA, USA).

## Results

A total of 98 women with spinal cord disease (SCD) were evaluated. Only three participants reported sexual orientation other than heterosexual (gay/lesbian; bissexual or other). Mean age was 43.0 (±12.1) years (19–69 years). The etiology of SCD was traumatic in 14 women (14.3%) and non-traumatic in 84 (85.7%), with multiple sclerosis as the most common cause, affecting 47 (55.9%) women. Regarding neurological impairment, 38 (38.8%) walked independently, 26 (26.5%) with an assistive device or orthosis and 21 (21.4%) were wheelchair users. Additional sociodemographic and clinical characteristics are summarized in Table 1.

**Table 1:** Sociodemographic and clinical characteristics of women with spinal cord disease.

	n%
**Marital Status**	
Single/Divorced/Widow	56 (57,1%)
Married/Stable Union	42 (42,9%)
**Education**	
High School	59 (60.2%)
College / Grad School	34 (34.7%)
Not Answered	5 (5.1%)
**Occupation**	
Employed	30 (30.6%)
Retired	38 (38.8%)
Unemployed	26 (26.5%)
Not answered	4 (4.1%)
**Cause of SCD**	
Multiple Sclerosis	47 (47.9%)
Traumatic	14 (14.2%)
Inflammatory or infectious diseases	12 (12.4%)
Disc herniation	8 (8.2%)
Tumor	3 (3,1%)
Other (myelomeningocele, etc)	14 (14.2%)
**Duration of spinal cord disease**	
≤ 5 years	21 (21.4%)
≥ 5 years	59 (60.2%)
Not Answered	18 (18.4%)
Neurological impairment*	
Walking independently	38 (38.8%)
Walking with assistive device/orthosis	26 (26.5%)
Wheelchair user	21 (21.4%)
Not Answered	13 (13.3%)
**Bladder management**	
Spontaneous voiding	65 (66.3%)
Clean intermittent catheterization	25 (25.5%)
Indwelling Catheter (urethral or suprapubic)	8 (8.2%)

Most participants (65 women, 66.3%) voided spontaneously, 25 (25.5%) performed clean intermittent catheterization (CIC), and 8 (8.2%) used an indwelling catheter (urethral or suprapubic). The mean NBSS-SF score was 11.0 (±6.9). Urinary incontinence was reported by 51 (52.0%) women, with severe incontinence in 28 (28.6%) of them. Satisfaction with bladder condition, however, was reported by only 34 (34.7%) women.

Sexual activity within the past six months was reported by 48 (49.0%) participants. The most frequently reported reasons for sexual inactivity were absence of a partner (48.0%), lack of interest (32.0%), and physical limitations (24.0%). The mean FSFI score was 22.0 (±10.1), with 28 sexually active women (58.3%) scoring ≤26.55, suggesting sexual dysfunction. Among these 48 sexually active women, 29 (60.4%) were satisfied with their sexual life. Notably, 9 sexually inactive women (18.0%) also reported satisfaction, highlighting the multifactorial nature of sexual well-being.

Sexually active women had lower NBSS-SF scores (mean difference −5.3, 95% CI − 8.0 to −2.6, p < 0.001). Among those with urinary incontinence, 37 (72.5%) were sexually inactive. Incontinent participants had lower FSFI scores than continent women (16.2 (10.3) vs. 24.4 (9.1); p < 0.001).

Univariate analysis identified younger age, marital status, premenopausal status, not being a wheelchair user, urinary continence, and not performing CIC as factors associated with sexual activity (Table 2). Being married significantly increased the likelihood of sexual activity (OR 12.8, 95% CI, 4.8 to 33.9; p < 0.001), while urinary incontinence was strongly associated with reduced likelihood of sexual activity (OR 0.12, 95% CI [0.0–0.3]; p < 0.001). Menopausal status was associated with sexual inactivity, with a higher proportion of postmenopausal women among sexually inactive than sexually active participants (44.0% vs 12.5%; p = 0.002). Wheelchair use was associated with a higher chance of sexual inactivity (28.0% vs 14.6%; p = 0.004).

**Table 2:** Predictors of sexual activity.

Predictors of sexual activity			
	Sexually active (n = 48)	Sexually inactive (n = 50)	P value
Age	38.0 ± 10.0	46.9 ± 12.6	< 0.001
Married/stable partner	34 (70.8%)	8 (16.0%)	<0.001
Menopause	6 (12.5%)	22(44.0%)	0.002
Mobility (wheelchair user)	7 (14.6%)	14 (28.0%)	0.004
Bladder management (CIC*)	7 (14.6%)	18 (36.0%)	0.015
Urinary incontinence	14 (29.2%)	37 (74.0%)	<0.001
Severe urinary incontinence	8 (16.6%)	20 (40.0%)	0.010

**Fig. 1:**
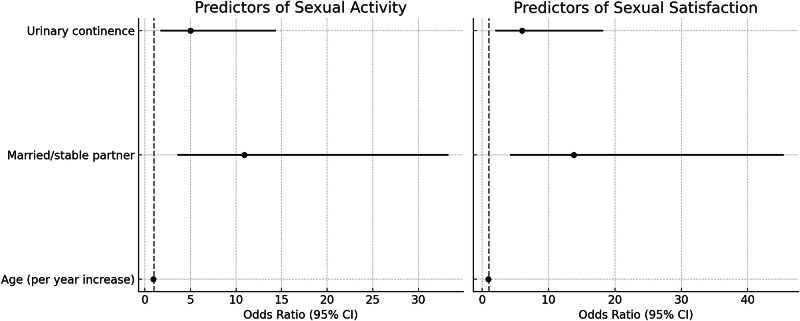
Forest plot showing adjusted odds ratios and 95% confidence intervals for the three multivariate predictors of sexual activity and sexual satisfaction, all of which were statistically significant (p < 0.05). Although the annual effect of age appears modest, it compounds over time to substantially lower the probability of sexual activity and satisfaction (see Fig. 2 for age-related trends).

**Fig. 2:**
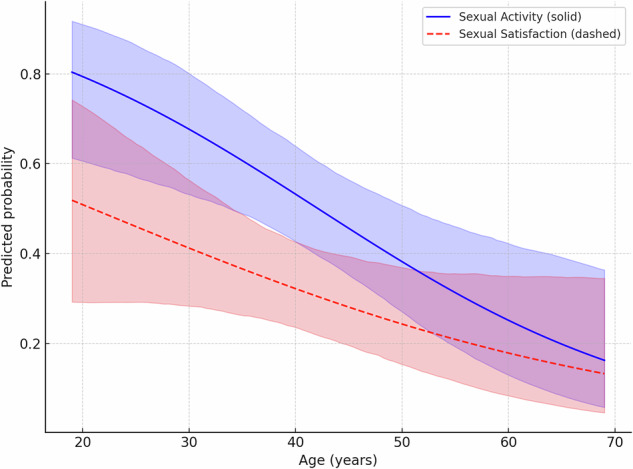
Probability of Sexual Activity and Sexual Satisfaction by Age in Women with Spinal Cord Disease: Predicted probability of sexual activity (solid line) and sexual satisfaction (dashed line) according to age, based on logistic regression models in women with spinal cord disease. The figure demonstrates a progressive decline in the likelihood of both outcomes as age increases, illustrating the cumulative impact of aging on sexual health in this population. Shaded areas indicate 95% confidence intervals.

On multivariate analysis, urinary continence (OR 5.0, 95% CI, 1.7 to 14.4; p = 0.012), younger age (OR 0.93, 95% CI, 0.89 to 0.98; p = 0.002), and marital status (OR 10.9, 95% CI, 3.6 to 33.3; p < 0.001) were significant predictors of sexual activity. Similarly, satisfaction with sexual life was associated with being married (OR 13.8, 95% CI, 4.2 to 45.4; p < 0.001), urinary continence (OR 6.0, 95% CI, 2.0 to 18.2; p < 0.001), and younger age (OR 0.93, 95% CI, 0.9 to 1.0; p = 0.009); see Table 3 and Fig. 1. Although the annual effect of age appears modest, it compounds over time to substantially lower the probability of sexual activity and satisfaction. Figure 2 demonstrates the cumulative impact of aging on sexual health in this population.

**Table 3:** Independent predictors of sexual activity and sexual satisfaction.

Variable	Sexual Activity OR (95% CI)	P value	Sexual Satisfaction OR (95% CI)	P value
Age*	0.93 (0.89–0.98)	0.002	0.93 (0.90–1.00)	0.009
**Married/stable partner**	10.9 (3.6–33.3)	<0.001	13.8 (4.2–45.4)	<0.001
**Urinary continence**	5.0 (1.7–14.4)	0.012	6.0 (2.0–18.2)	<0.001

## Discussion

This study reveals a substantial burden of sexual dysfunction and urinary incontinence among women with SCD. Sexual dysfunction and urinary incontinence are both common and multidimensional in this population, with nearly 60% of sexually active women scoring in the dysfunctional range on the FSFI and over half of participants reporting urinary incontinence, which was strongly associated with reduced sexual activity and satisfaction. Although our data show high prevalence of bladder symptoms and sexual inactivity and their associations, they do not allow causal inference regarding the effects of UI treatment. Younger age, marital status, and urinary continence were independent predictors of both sexual activity and sexual satisfaction, demonstrating the combined influence of physical, relational, and psychological factors on sexual health after SCD.

These results are consistent with prior research. Elmelund et al [12]. reported urinary incontinence in 49% of women with neurological disease, closely matching our finding of 52% with nearly one-third experiencing severe symptoms. Mahler et al. [23] described pervasive concerns about urine leakage during intimacy in women performing intermittent catheterization, a concern mirrored in our cohort, where urinary incontinence independently predicted sexual inactivity and lower FSFI scores. The association between clean intermittent catheterization and reduced sexual activity further illustrates the impact of bladder dysfunction on sexual well-being [11, 12, 15]. Embarrassment, diminished self-confidence, and fear of leakage can impact sexual spontaneity and contribute to the avoidance of intimacy [8, 12]. Although behavioral, pharmacologic, and surgical treatments for urinary incontinence are available, these issues remain under-addressed among women with neurological disease [14, 24]. Evidence from women with non-neurogenic urinary incontinence suggests that treatment with intravesical onabotulinumtoxinA [25], sacral neuromodulation [26] or pelvic floor exercises [27] can improve sexual function and satisfaction, which provides a rationale for exploring similar approaches for neurogenic cases.

Despite the central role of sexual health in quality of life, sexuality and intimacy are frequently overlooked in SCD care. Studies indicate that healthcare providers often avoid discussions of sexual function due to discomfort, limited training, or the perception that sexuality is peripheral to neurological rehabilitation [2, 5, 28]. Our findings underscore the importance of addressing sexual health systematically as part of routine clinical care.

Besides urinary continence, marital status, and younger age were associated not only with increased sexual activity, but also with greater sexual satisfaction. These associations reflect the importance of functional and relational factors in shaping both behavioral and subjective aspects of sexual well-being [9, 24].

Several limitations of this study should be considered. First, it was conducted in a single tertiary rehabilitation centre with specific interdisciplinary resources and established routines for LUTS and sexual counselling. As such, our findings may not be fully generalisable to settings with different levels of rehabilitation infrastructure or access to specialised urological and sexual health care.

Second, the cross-sectional design, without any intervention or post-treatment reassessment precludes causal inference. Third, the FSFI was originally validated for women with recent sexual activity within a 4-week window, and its standard scoring and interpretation are not optimized for women with very infrequent or absent partnered sexual activity, nor does it fully address broader relational and psychosocial aspects of sexual wellbeing. In the present study, we used a broader 6-month window because many women with SCD report infrequent partnered sexual encounters; applying a strict 4-week criterion would have excluded a substantial proportion of sexually active women and reduced the clinical relevance and external validity of the findings. We acknowledge that this approach may increase recall bias. Furthermore, no universally accepted, formally validated scheme exists for stratifying total FSFI scores into mild, moderate, or severe dysfunction; for this reason, we used the ≤26.55 cut‑off only as a dichotomous screening indicator and focused our primary analyses on sexual activity and global satisfaction with sexual life rather than on FSFI severity grading. Moreover, the FSFI may underestimate the burden of dysfunction among sexually inactive women, as its standard scoring is not validated for women with very infrequent or absent partnered sexual activity and does not fully capture satisfaction or distress outside the context of partnered sexual activity. Notably, some sexually inactive women in our sample reported satisfaction with their sexual life, suggesting that sexual wellbeing may be supported by emotional intimacy, non-coital sexual expression, or adaptive perspectives (e.g. not missing sexual activity or feeling relieved from potential difficulties associated with sexual encounters) [6, 24].

Important confounders - including mood disorders, body image concerns, partner availability, and history of sexual trauma - were also not assessed and may have influenced results. We also did not assess incontinence specifically during sexual activity or fecal incontinence, both common in this population and potentially impactful on sexual health.

Finally, the use of mobility status as a surrogate for neurological impairment, while practical, does not reflect key dimensions such as perineal/genital sensory loss or specific motor limitations that may affect sexual positioning, balance, and the ability to maintain certain positions during intercourse. Thus, women categorized as “mobile and independent” may still experience profound sensory impairment or functional restrictions with major consequences for sexual arousal, orgasm, and satisfaction.

While sexual health is central to emotional wellbeing and quality of life for women with SCD, yet sexual dysfunction remains common and under-recognized in this group. This study identifies strong associations between urinary incontinence, sociodemographic factors, and sexual outcomes, and underscores the need for further investigation using more comprehensive neurological and functional assessments to address this multifaceted issue in greater depth. Although our findings do not allow us to conclude that treating UI will necessarily improve sexual or emotional health, they do support current rehabilitation recommendations that call for systematic screening and counselling on urinary and sexual health in women with SCD, particularly in those with urinary incontinence. Recognising these symptoms and addressing them proactively in rehabilitation programs may help improve sexual activity and overall quality of life in this population. Further interventional and qualitative research is needed to determine whether targeted management of urinary incontinence can enhance sexual health and quality of life among women with SCD.

## Data Availability

The datasets generated and/or analyzed during the current study are included within the published article. Additional data are available from the corresponding author on reasonable request.
